# Supplementary material for: Leukocyte Telomeric G-Tail Length Shortening Is Associated with Esophageal Cancer Recurrence
Source: J Clin Med. 2022 Dec 12;11(24):7385. doi: 10.3390/jcm11247385 (PMC9784295; doi:10.3390/jcm11247385)
Supplement: Supplementary file 1 [file jcm-11-07385-s001.zip › jcm-2034795-supplementary.pdf]

Supplementary Figure

S1 A

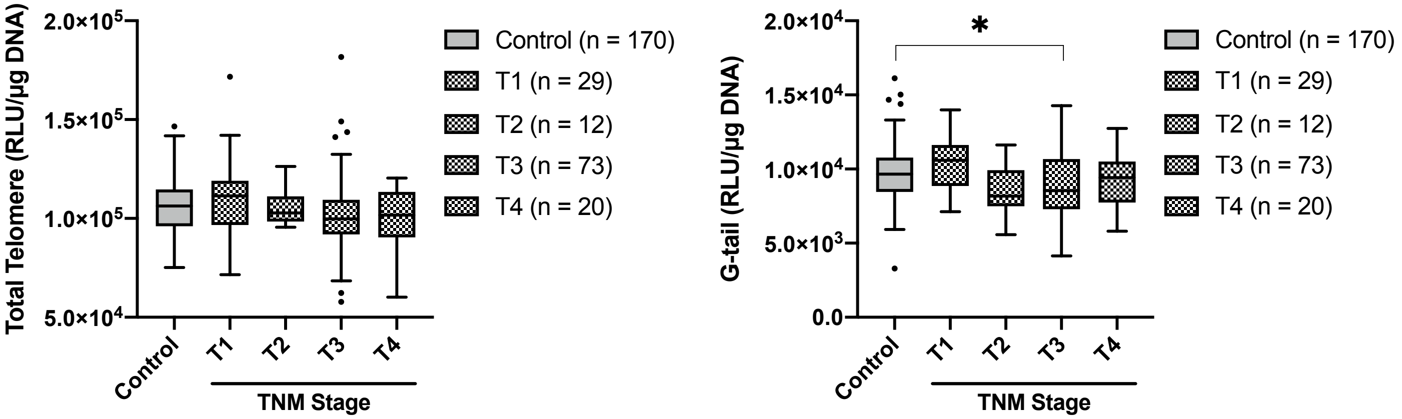

B

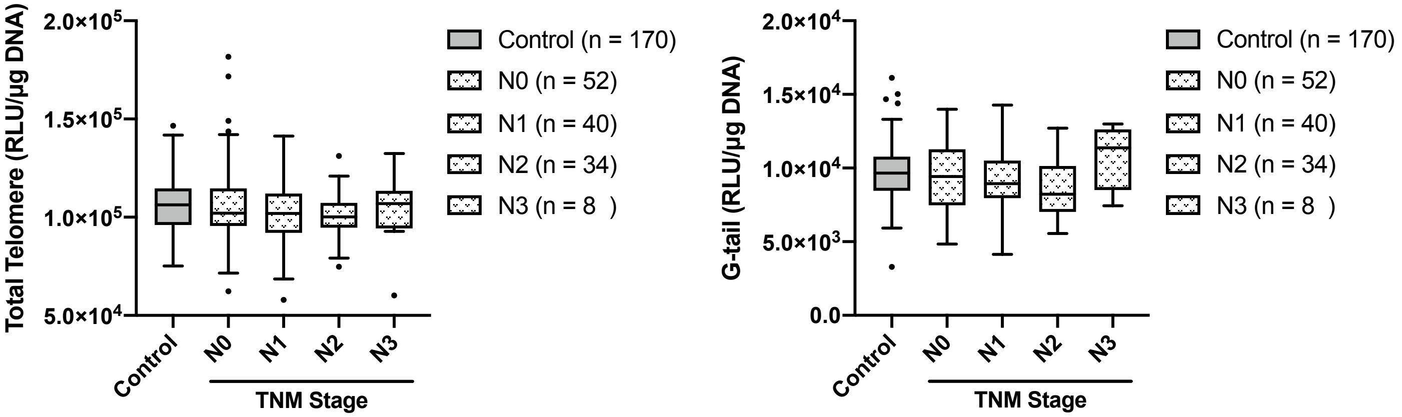

C

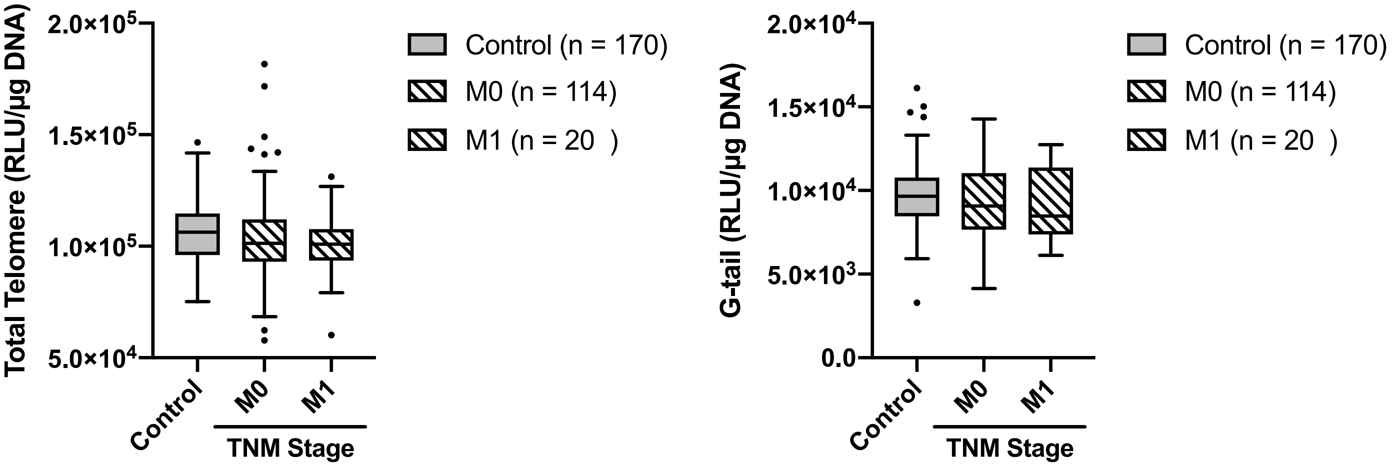

Supplementary Figure S1. Comparison of LTL/G-tail length in controls and patients with ESC in T, N, M subsets, respectively. (**A**) Box-and-whiskers plot representing the comparison of LTL (left panel) and G-tail length (right panel) between controls and patients with ESC in T subsets. (**B**) Box-and-whisker plots representing the comparison of LTL (left panel) and G-tail length (right panel) between controls and patients with ESC in N subsets. (**C**) Box-and-whiskers plot representing the comparison of LTL (left panel) and G-tail length (right panel) between controls and patients with ESC in M subsets. Error bars indicate the standard error of the mean. * represents significant difference ($0.01 < p < 0.05$).
